# Effects of transient high temperature treatment on the intestinal flora of the silkworm *Bombyx mori*

**DOI:** 10.1038/s41598-017-03565-4

**Published:** 2017-06-13

**Authors:** Zhenli Sun, Dhiraj Kumar, Guangli Cao, Liyuan Zhu, Bo Liu, Min Zhu, Zi Liang, Sulan Kuang, Fei Chen, Yongjie Feng, Xiaolong Hu, Renyu Xue, Chengliang Gong

**Affiliations:** 1 School of Biology & Basic Medical Science, Soochow University, Suzhou, 215123 China; 2 National Engineering Laboratory for Modern Silk, Soochow University, Suzhou, 215123 China; 3 Agricultural Biotechnology Research Institute, Agricultural biotechnology and Ecological Research Institute, Soochow University, Suzhou, 215123 China

## Abstract

The silkworm *Bombyx mori* is a poikilotherm and is therefore sensitive to various climatic conditions. The influence of temperature on the intestinal flora and the relationship between the intestinal flora and gene expression in the silkworm remain unknown. In the present study, changes of the intestinal flora at 48, 96 and 144 h following transient high temperature treatment (THTT) of 37 °C for 8 h were investigated. According to principal component analysis, the abundances of *Enterococcus* and *Staphylococcus* showed a negative correlation with other dominant genera. After THTT, the gene expression levels of spatzle-1 and dicer-2 were increased and decreased, respectively, which suggested that the Toll and RNAi pathways were activated and suppressed, respectively. The species-gene expression matrix confirmed that the spatzle-1 and dicer-2 gene expression levels were negatively and positively correlated, respectively, with the abundance of *Enterococcus* and *Staphylococcus* in the control. The abundance of *Variovorax* post-THTT was positively correlated with the spatzle-1 gene expression level, whereas the community richness of *Enterococcus* was negatively correlated with the spatzle-1 gene expression level and positively correlated with the dicer-2. The results of the present investigation provide new evidence for understanding the relationships among THTT, intestinal flora and host gene expression.

## Introduction

The silkworm *Bombyx mori* (Lepidoptera) is a poikilotherm that is highly sensitive to environmental temperature due to artificial domestication and indoor rearing. The most suitable temperature for silkworm development is approximately 20–30 °C. It is well known that genetic traits, economic traits^1^ and pathogen resistance^2^ of silkworms are tightly linked to the surrounding temperature. Previous studies have indicated that gene expression profiles of silkworms are changed after exposure to high temperature^3, 4^. Comparative proteomic analysis of the silkworm posterior silk gland has shown that high temperature alters the expression of proteins related to silk synthesis^5^. A report on the effect of temperature on silkworm hemocyte cell cycle revealed that high temperature induces G2 arrest in larval hemocytes^6^.

The gut flora plays a crucial role in the development and environmental adaptation in the host insect^7^, and the microbiota has an extensive effect on host physiology, ranging from immunity to gut structure^8^. Recent research has indicated that the social attraction of *Drosophila* is mediated by its microbiome^9^ and that there is a close relationship between animal behavior and the microbiome^10^. It has also been reported that dysbiosis or dysbacteriosis of the gut flora is strongly associated with the age-onset intestinal barrier dysfunction in *Drosophila* ^11^ and that the gut flora plays a significant role in maintaining immune homeostasis in the fly gut^12^. Moreover, the germ line of *D*. *melanogaster* is influenced by gut bacteria, and the main impact on oogenesis is related to the lack of *Acetobacter* ^13^. Both commensal and pathogenic intestinal microbes have shown influence on *Drosophila* carbohydrate and lipid metabolism, and the host metabolism has been shown to be regulated by metabolites generated through gut microbes^14^. The abundance of intestinal flora also depends on the host genotype, which can alter nutrient allocation patterns^15^, and the microbiota can affect the host nutritional status by adjusting nutrient acquisition^16^. Additionally, host metabolic gene expression can be modulated by the *Drosophila* microbiota via IMD/NF-κB signaling and expression of genes involved in host digestive functions, and primary metabolism is also promoted by the microbiota^17^.

The silkworm *Bombyx mori* is an economically important domesticated insect, and the economic traits of silkworm are closely related to the silkworm strain, rearing environment and quality of mulberry leaf. It is well known that nutrient absorption, nutrient utilization, and silkworm diseases are directly linked to gut flora of silkworm larvae^18^. Therefore, information about the relationships among gut bacteria and surrounding factors can contribute to the improvement of the health and nutrient absorption of silkworms.

The silkworm gut microbiota and its composition are impacted by the silkworm strain and forages and also reflects silkworm health. In silkworms infected with *Nosema bombycis*, the number of bacterial species of the genus *Enterococci* is decreased compared to healthy silkworms, while the number of bacteria of the genus *Enterococci* is increased^19^. We previously reported that gut flora of the silkworm change with developmental stage and gender. The composition and diversity of gut flora are noticeably decreased at the later stage of the fifth instar larvae. The community richness of *Delftia*, *Aurantimonas* and *Staphylococcus* is lower in male silkworms than in females, whereas the abundance of *Enterococcus* is greater in males. The diversity of gut flora in silkworms infected with *B*. *mori* cypovirus (BmCPV) is decreased, but the abundance of both *Enterococcus* and *Staphylococcus* is increased^20^. To date, the effects of temperature on economic traits^1^, pathogen resistance^2^ and development have been well studied. The impact of temperature on silkworm gene expression has been reported by several researchers^3, 4^, but little progress has been made in understanding the influence of temperature on intestinal flora in silkworms and the relationships among temperature, intestinal flora and gene expression. In the present study, we explored the influence of transient high temperature treatment (THTT) on intestinal flora and gene expression in the silkworm *B*. *mori*. Our findings revealed that after THTT, the diversity of intestinal flora was increased, Toll signaling pathways were activated and RNAi signaling pathways were suppressed. These changes may be attributed to the alterations and relationships among temperature, intestinal microbiota and host gene expression.

## Material and Methods

### High temperature treatment and collection of intestinal contents

*B*. *mori* silkworm larvae (Daizo strain) were reared at 25 °C on fresh mulberry leaves after hatching in a rearing room. The newly molted fifth instar larvae were exposed to 37 °C for 8 h, and all exposed larvae were then reared at normal temperature (25 °C) till the end of fifth instar. The midguts of 30 larvae from each treatment were dissected in a sterile environment at 48, 96 and 144 h post-THTT. The collected intestinal contents were immediately frozen and stored at −80 °C. A separate control batch of silkworms was reared at normal temperature (25 °C) without exposure to 37 °C during the same time. One sample was sequenced twice, and both sequenced data were analyzed and used to perform additional experiments. Collected samples were named with the convention of CT/HT-N with CT representing control sample, HT representing THTT sample and N representing the sample collected at N h post-THTT.

### Sample preparation

The collected intestinal contents were mixed with sterile water, filtered with a nylon net bag (TPE-200 mesh-03A, Shanghai Hengtai Filtering Equipment Co., Ltd., Shanghai, China) and washed with 500 ml of sterile water. Liquid was filtered using a fibroid membrane (aperture 0.45 μm, Xinya Purification Device Factory, Shanghai, China), and filter membranes were stored at −80 °C for further experiments.

### DNA extraction, amplification, purification and sequencing

DNA extraction, amplification and purification were performed according to our previous study^20^. Briefly, DNA from filtered membranes was assessed with 1% agarose gels and extracted using a DNA extraction Kit. DNA concentration was determined using a Qubit® 2.0 fluorometer (Life Technologies, CA) and diluted to 10 ng/μL. To perform Illumina miseq sequencing, PCR products representing the V1-V3 region of 16 S rRNA genes were amplified using the following universal primers: 341F-91 (CCTACACGACGCTCTTCCGATCTNCCTACGGGNGGCWGCAG) and Miseq-805R (GACTGGATTCCTTGGCACCCGAGAATTCCAGACTACHVGGGTATCTAATCC). The PCR products were purified and quantified, and the concentration was adjusted to 25 ng/μL. An equal amount of each sample was mixed, and sequencing was performed with an Illumina miseq (PE300) sequencing system. Sequenced data was assigned to the corresponding sample according to barcode sequences.

The mate pairs from fragments shorter than twice the length of the reads were overlapped and merged by Flash software (FLASH v1.2.7) to increase the length of the reads. Preclustered commands with permitted max mismatches less than 1/150 (http://www.mothur.org/wiki/Pre.cluster) were used to screen and remove noise to obtain unique sequences. UCHIME in Mothur software (http://www.mothur.org/) was used to remove chimera sequences.

### Taxonomical classification

After clustering unique sequences into operational taxonomic units (OTUs) defined at the 97% similarity threshold (http://www.drive5.com/uclust/downloads1_1_579.html), the OTU-representative reads were assigned to genus using Mothur’s version of the Ribosomal Database Project (RDP) Bayesian classifier through a normalized RDP training dataset^21^.

### Evaluation of richness and diversity

Alpha diversity and species richness were estimated using the Mothur software^22^ and rarefaction curves^23^, respectively. Bacterial diversity in the sample was evaluated with Shannon–Wiener and Simpson diversity indexes^24^. The total number of species (an exponent of the OTU number) in samples was estimated with Chao1^25^ and Ace^26^ [S_chao1_ = S_obs_ + n1(n1 − 1)/2(n2 + 1), where S_obs_ is the observed number of species, n1 is the number of OTUs with only one sequence, and n2 is the number of OTUs with only two sequences]. The authenticity of the sequenced data was evaluated with coverage^27^.

### qRT-PCR

The relative expression level of genes related to innate immunity, cell cycle and apoptosis were determined by qRT-PCR according to our previous methodology^28^. The primers used for qRT-PCR are listed in Table 1. Relative gene expression normalized to the actin A3 gene was estimated according to the 2^−∆∆Ct^ method^29^.

**Table 1:** The primers used in this study.

Genes	Primers	Sequences
spatzle-1 (Bmspz)	forward	CAGGATTCGCCTCACAGTCAC
	reverse	CAGTTCGGGATGCTTCCTCGAT
peptidoglycan-recognitionprotein LB (BmPGRP-LB)	forward	GAACAGTGCAGTAGCCGCCATG
	reverse	CCTCGATCGGTCCATCCTCG
peptidoglycan-recognition protein LE (BmPGRB-LE)	forward	CACTGCAACAGAAAGCTGTAG
	reverse	CGCAATATGCCGATCCGTCAC
Suppressor of cytokine signaling (SOCS2)	forward	GTGACAGACCGTTGGCTAGG
	reverse	GCACCGGCGAGTGTGGACAC
signal transducer and activator of transcription (BmSTAT)	forward	GAGCGTTATGGACGAGAAGC
	reverse	CCTGGTTGCCGTGGACTATG
dicer-2 (Bmdrc-2)	forward	CCAGCGTTCACGTCCGTTGGA
	reverse	GATCGCCAAGATTTGGTCGAA
*Actin3*	forward	AACACCCCGTCCTGCTCACTG
	reverse	GGGCGAGACGTGTGATTTCCT
*OVO protein 1* (Bmovo)	forward	GCCCCTTACCGCTCCTTTGG
	reverse	ATCGCCTCCAAGAATCGATG
*Decapentaplegic* (Bmdpp)	forward	GCACGTACCGACGGAGCTCG
	reverse	GAGATCAGCACCACGAGCTAC
Yorkie (Bmyki)	forward	GGCCACGACAGCGGAAGG
	reverse	TCGCCAAAGACGATACGATAGAAGA
*Expanded* (Bmex)	forward	CGAGGCATGACCGATACGGA
	reverse	GCATCTAATCCATGAGTGTG
*Cyclin E* (Bmcyc-E)	forward	GACGTTCCATATCTGGTGCATG
	reverse	GGAGACCCAGATTCAGCCATA
inhibitor of apoptosis protein (Bmiap)	forward	TTGCAAGACGAGTGTCAGTG
	reverse	CACCAACGTGGCGGCAGCTCC
c-myc (BmMyc)	forward	CCGACCGCATATTCTCAGTGAG
	reverse	ATGGGTTCAACGCACACCGTC
*Delt* (*Bmdelt*)	forward	CTAGACAACGACTACGGGTG
	reverse	CTGTACCCGTCCACTCGGTC
tarsal-less (Bmtal)	forward	CGGTCTCTATTAACCATGGAGC
	reverse	CACATGTTGACATCAGTACAC

### Relevance analysis between samples

The phylogenetic tree was inferred using the Maximum Likelihood method with MEGA6.0^30^. Principal coordinate analysis (PCoA) was used to compare bacterial community structures based on weighted-UniFrac from each library using R software (R Development Core Team, 2009, http://www.r-project.org). The differences between bacterial communities from each sample were compared using analysis of molecular variance (AMOVA) in Mothur based on weighted-UniFrac distances^22^. Correlation between dominant genera was analyzed with principal components analysis (PCA) with linear ordination methods using CANOCO 4.5^31^.

−∆Ct values from qRT-PCR were used for gene expression-species matrix analysis. After receiving spe.dta and env.dta, files were generated using the WCanoImp program of Canoco for Windows 4.5 software package, and detrended correspondence analysis (DCA) was then performed. Canonical correspondence analysis (CCA) or redundancy analysis (RDA) was used for ordination according to gradient lengths to address the relationship between microbial communities and gene expression level. Venn diagrams were created with the Venny online tool (http://bioinformatics.psb.ugent.be/webtools/Venn/).

## Results

### Analysis of the Illumina miseq sequencing-derived dataset

**Figure 1:**
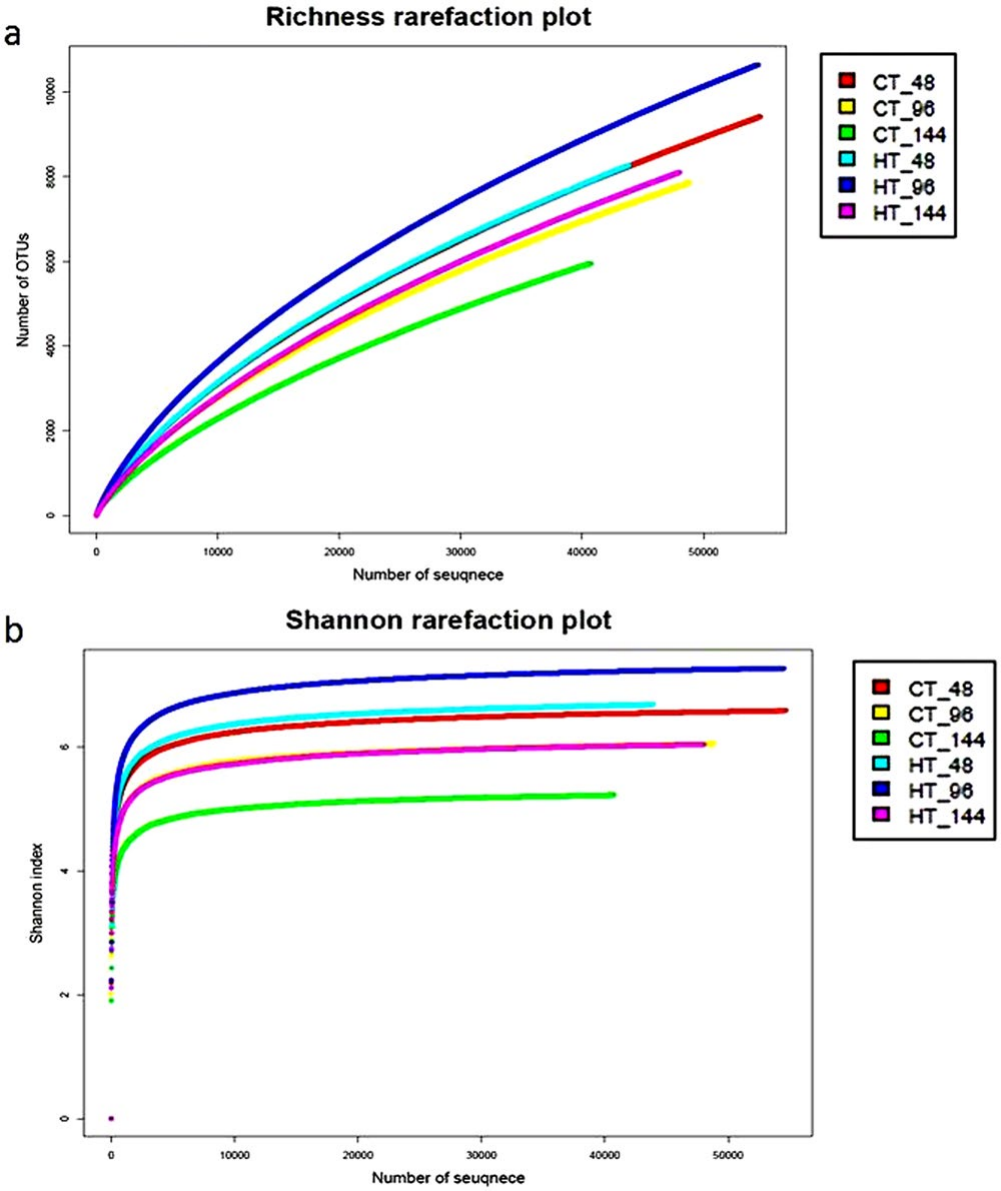
Richness rarefaction and Shannon index analysis of the different samples. CT-48 (96, 144) and HT-48 (96, 144) are samples mentioned in Table 1. (**a**)Rarefaction curves of OTUs clustered at 97% sequence identity across difference samples. (**b**) Rarefaction curves of the Shannon index according to OTU.

The original data of Illumina miseq sequencing related to this article is available in GenBank (Table S1). After removal of low-quality reads, 144,206 and 146,315 valid reads were obtained from the control and THTT samples, respectively, using Illumina miseq sequencing of 16 S rRNA genes. The number of reads varied in different samples (Table 2). Sampling coverage was used to evaluate the authenticity of sequenced data, and the results showed that the coverage was 0.899 to 0.911 (Table 2), indicating that the sequenced data were credible. Sequencing data were used to assess the diversity and richness of gut bacteria. The richness rarefaction curves for individual samples did not tend to approach the saturation plateau (Fig. 1a), indicating the true bacterial richness in the gut content was underestimated. The Shannon index was used to estimate the bacterial diversity, and the Shannon rarefaction curves tended to plateau (Fig. 1b). The Shannon index of the gut microbiota of control silkworm was slightly lower than THTT silkworms. The results revealed that the bacterial species diversity in the gut content of control silkworms was less than that of THTT silkworms, and a similar trend was noticed in the Simpson index analysis. The OTU number of a bacterial community was estimated by Chao1 and Ace, and the results indicated that the total number of species of the silkworm gut content depended on the developmental time or maturity of the fifth instar silkworm larvae (48, 96 and 144 h). Community richness in the HT-48 sample was lower than that of CT-48, while the abundance in HT-96 and HT-144 samples was greater than that in CT-96 and CT-144 samples, respectively, according to Chao1 and Ace (Table 3), suggesting that the silkworm gut flora was influenced by the exposure to transient high temperature.

**Table 2:** Composition of microbial communities in the control and THTT silkworm samples.

Sample	valid reads	Phylum	Class	Order	Family	Genus	Species
CT-48	54653	28	62	122	242	796	695
CT-96	48850	27	60	117	233	760	642
CT-144	40703	29	59	112	217	609	477
Total CT	144206	29	64	130	261	1001	1019
HT-48	43853	31	61	122	245	834	684
HT-96	54461	32	65	128	248	925	846
HT-144	48001	29	60	116	233	746	653
Total HT	146315	34	68	133	265	1130	1272

Collected samples were named with the convention CT/HT-N with CT representing control sample, HT representing high temperature treatment sample and N representing the sample was collected at N h post- high temperature treatment.

**Table 3:** Richness and diversity indexes in all samples.

indices	48 h		96 h		144 h	
	CT	HT	CT	HT	CT	HT
No. of OTU	8715	7298	8189	9297	6400	6802
Shannon	6.438	6.535	6.146	7.028	5.339	5.765
ACE	29695.83	21890.01	26604.54	28508.96	24056.51	25398.49
Chao1	19712.72	15300.47	17202.31	19650.28	15536.07	16051.5
Simpson	0.022	0.021	0.044	0.012	0.099	0.033
Coverage	0.904	0.904	0.900	0.902	0.899	0.911

CT/HT was sample mentioned in Table 1. Chao1 and ACE indexes were used to estimate the total number of species in samples. Bacterial diversity was assessed with Shannon–Wiener diversity index and Simpson index, Shannon–Wiener diversity index positive correlation with microbial diversity, Simpson index negative correlation with microbial diversity.

### Effects of THTT on silkworm intestinal microbiota

The OTU-representative reads for individual samples in the control and THTT silkworm gut contents (THTT values shown in parentheses) were assigned to the different levels of taxonomical classification as follows: 29 (34) phyla, 64 (68) classes, 130 (133) orders, 261 (265) families, 1001 (1130) genera and 1019 (1272) species. In general, the results showed different bacterial diversity between CT and THTT samples, with the highest diversity present in the THTT group. However, among these THTT groups (HT-48, HT-96 and HT-144), the number and bacterial diversity at different classification levels were highest in the HT-96 sample (Table 2). In individual THTT samples, the number of bacteria recorded at the genus level was higher than that recorded at the species level, suggesting that some identified bacteria could not be classified into species based on 16 S rRNA gene sequencing data (Table 2).

With regard to bacterial species, the highest number of unclassified species was observed in the HT-48 sample compared to all THTT and control groups, indicating that unclassified bacteria species were increased in the HT-48 sample. In the HT-48, HT-96 and HT-144 samples, the number of various types of bacteria at the genus (species) level were 38 (−11), 165 (204) and 137 (176), respectively, more than the control samples (Table 2) with the negative sign indicating that the number of bacteria at the species level was less than the corresponding control.

The differences in the composition of gut flora expressed as bacterial abundance increased from 48 h post-treatment, peaked at 96 h post-treatment, then decreased and reached a normal stage. These data indicated that the effect of THTT on the gut flora was decreased after the THTT silkworms were reared at normal temperature (25 °C) for a period of time.

**Figure 2:**
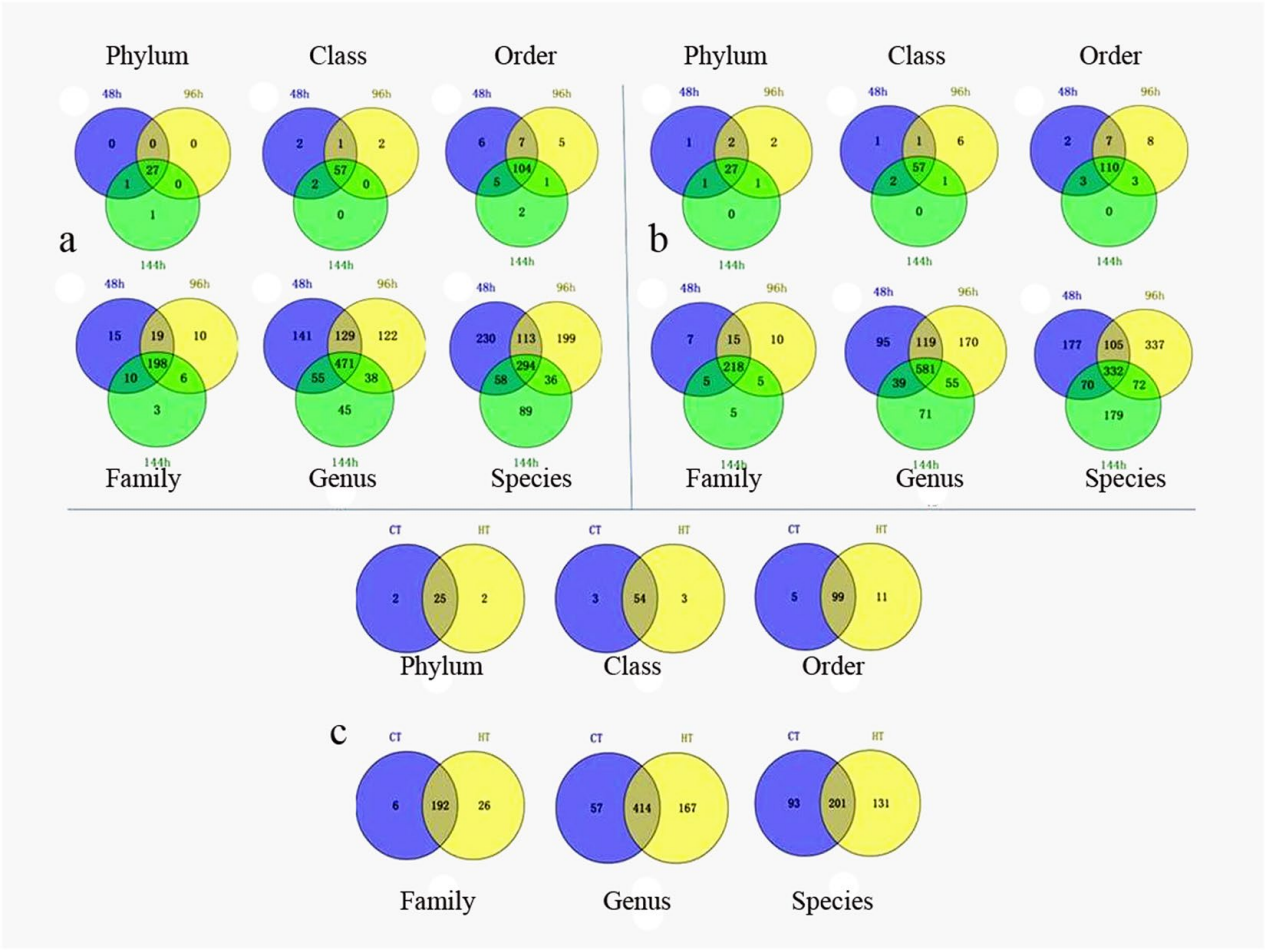
Shared bacterial types of different samples at different classification levels. (**a**) Venn analysis of the control silkworms at different time points; (**b**) Venn analysis of THTT silkworms at different time points; (**c**) Venn analysis of control and THTT silkworms. CT, control silkworms; HT, silkworms exposed to transient high temperature; 48, 96 and 144 h, silkworms exposed to transient high temperature followed by rearing at normal temperature for 48, 96 and 144 h, respectively.

Venn analysis showed that 27 (27) phyla, 57 (57) classes, 104 (110) orders, 198 (218) families, 471 (581) genera and 294 (332) species were shared by the control (THTT) samples (Fig. 2a,b), and 25 phyla, 54 classes, 99 orders, 192 families, 414 genera and 201 species were detected in the all samples (Fig. 2c), suggesting that these bacteria were the basic bacteria of the silkworm gut flora.

At the phylum level, both Crenarchaeota and Fibrobacteres were absent in all THTT samples, while Elusimicrobia and Caldiserica were only present in the THTT samples, indicating that Crenarchaeota and Fibrobacteres were omitted from the silkworm gut content due to sensitivity towards high temperature. At the class level, Thermoprotei, Fibrobacteria, Proteobacteria and Incertae_Sedis were absent in THTT samples, while Elusimicrobia, Caldisericia and Dehalococcoidia were present only in THTT samples. Overall, 5 orders, 6 families, 57 genera and 93 species disappeared, but 11 orders, 26 families, 167 genera and 131 species were newly recorded after silkworms were subjected to THTT. Therefore, we revealed that intestinal bacterial diversity increased with THTT (Fig. 2c).

**Figure 3:**
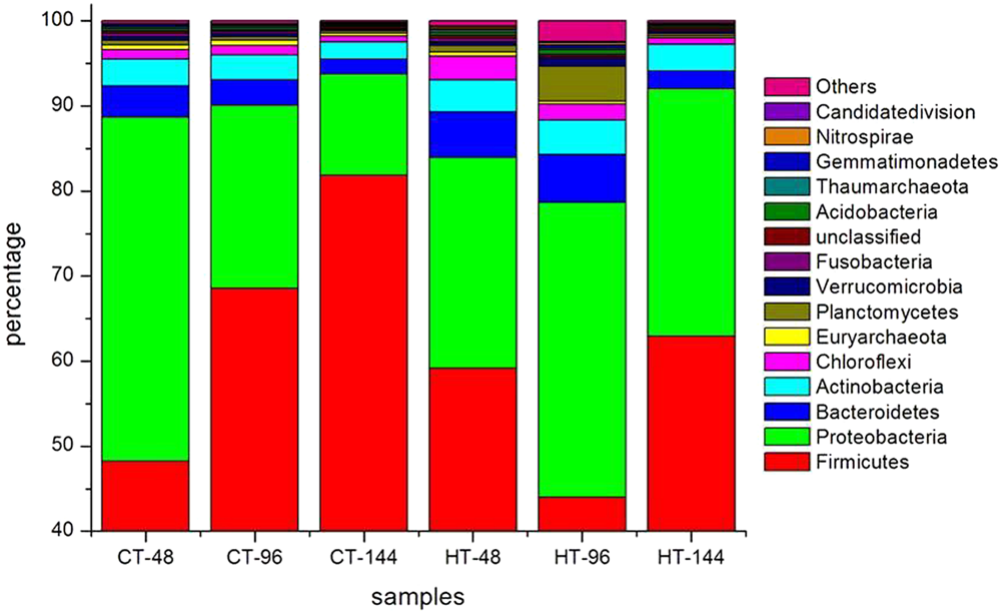
The dominant phyla percentage for all samples at each time point. CT/HT-N is sample mentioned in Table 1.

The predominant phyla and its abundance in the individual samples are shown in Fig. 3. In the HT-48, HT-96 and HT-144 samples, the predominant phyla were Firmicutes (59.17%, 43.98%, and 62.95%), Proteobacteria (24.77%, 34.65%, and 29.11%), Bacteroides (5.39%, 5.67%, and 2.06%), Actinobacteria (3.73%, 4.05%, and 3.17%), Chloroflexi (2.77%, 1.84%, and 0.79%), Planctomycetes (0.71%, 4.16%, and 0.31%), Cyanophyta (0.15%, 1.96%, and 0.07%), Euryarchaeota (0.53%, 0.33%, and 0.16%), Verrucomicrobia (0.43%, 0.87%, and 0.26%), Fusobacteria (0.41%, 0.22%, and 0.22%), unclassified (0.39%, 0.29%, and 0.19%), Acidobacteria (0.31%, 0.55%, and 0.17%), Gemmatimonadetes (0.18%, 0.37%, and 0.09%), Thaumarchaeota (0.28%, 0.17%, and 0.14%) and Nitrospira (0.15%, 0.29%, and 0.05%) (Fig. 3). The richness of Firmicutes initially decreased and then increased with maturity of the fifth instar silkworm larvae. Changes in the abundance of Proteobacteria, Bacteroides, Actinobacteria, Planctomycetes, cyanophyta, Verrucomicrobia, Acidobacteria, Gemmatimonadetes and Nitrospira were contrary to that of Firmicutes. The abundance of Chloroflexi, Euryarchaeota, Fusobacteria, Thaumarchaeota and unclassified bacteria decreased as the larvae matured.

The abundance of all identified bacterial phyla in the THTT samples was compared to the corresponding controls. The abundance of 11 phyla (*p* < 0.05 and q < 0.05) was significantly different in CT-48 and HT-48 samples. However, 16 phyla (*p* < 0.05 and q < 0.05) were recorded in CT-96 and HT-96 samples, and 7 phyla (*p* < 0.05 and q < 0.05) were observed in CT-144 and HT-144 samples (data not shown). In comparison to the main predominant phyla of THTT and control samples, the percentage of Firmicutes in HT-48 was 1.23 times higher than the corresponding control, whereas the percentages of Firmicutes in HT-96 and HT-144 were 0.64 and 0.77 times lower, respectively, than the corresponding controls. The percentages of Proteobacteria (Actinobacteria) in the HT-48, HT-96 and HT-144 samples were 0.61 (1.20), 1.61 (1.39), and 2.45 (1.54) times higher, respectively, than the corresponding controls. The abundance of Planctomycetes in the HT-96 sample was 8.85 times higher than the control sample, and a similar trend was present for other bacterial phyla in the HT-96 sample. These outcomes suggested that the microbiota is sensitive to high temperature.

In THTT samples, the abundance of all identified bacterial genera was compared to the corresponding control. The abundance of 54 genera (*p* < 0.05 and q < 0.05) was significantly different in the CT-48 and HT-48 samples. However, 202 genera (*p* < 0.05 and q < 0.05) were observed in the CT-96 and HT-96 samples, and 29 genera (*p* < 0.05 and q < 0.05) were recorded in the CT-144 and HT-144 samples (data not shown). These results indicated that the influence of THTT on intestinal bacterial flora was delayed and that the effect of THTT decreased with the passage of time.

**Figure 4:**
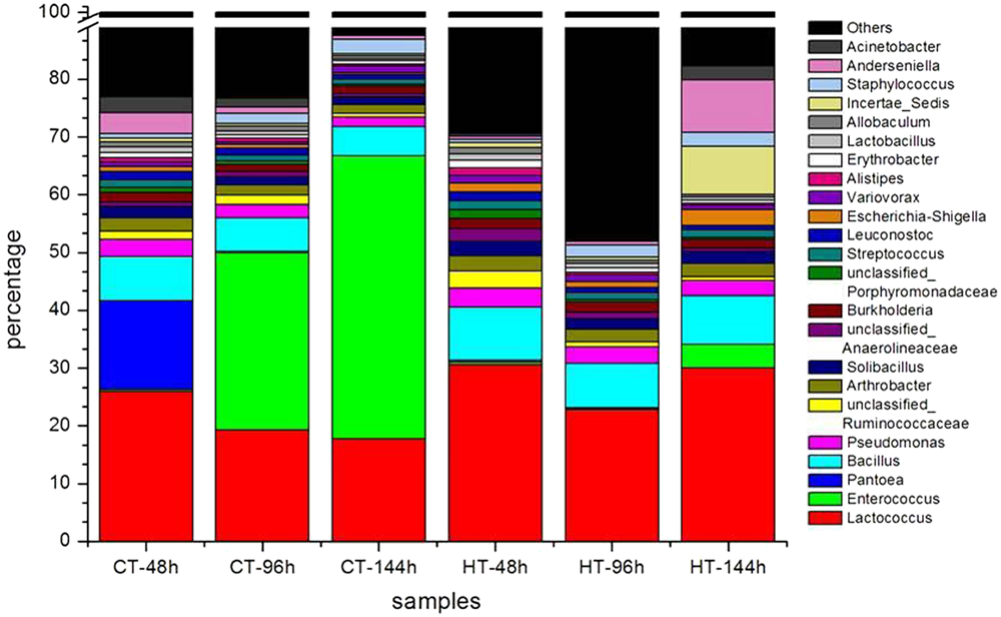
The dominant genera percentage for all samples at each time point. CT/HT-N is sample mentioned in Table 1.

The dominant genera and their abundance in all samples are shown in Table 4 and Fig. 4. The abundance of *Enterococcus* in the HT-48 and CT-48 samples was similar to the corresponding controls, but the abundance of *Enterococcus* in the HT-96 and HT-144 samples was 133.4 and 12.18 times lower, respectively, than the corresponding controls. Therefore, we hypothesized that the abundance of *Enterococcus* in the silkworm gut content was significantly affected and sensitive to high temperature. However, other bacterial genera were also affected by high temperature but not to the same extent as *Enterococcus* (Table 4). After treatment, the THTT silkworms were reared at normal temperature (25 °C) for a period of time, and the influence of high temperature decreased as the silkworm larvae matured or as time passed. In the HT-48 sample, the abundance of *Pantoea*, *Anderseniella* and *Acinetobacter* was decreased by 52.55, 6.54 and 5.96 times, respectively, compared to the control. The richness of *Acinetobacter* and *Anderseniella* in the HT-96 sample was 4.93 and 1.81 times lower, respectively, than the control sample. In the HT-144 sample, the abundance of *Acinetobacter*, *Anderseniella*, *Escherichia*-*Shigella* and *Incertae*_*Sedis* was 9.76, 15.54, 7.71 and 26.87 times higher, respectively, than the control sample. These results indicated that the silkworm gut flora was easily affected by the environmental temperature and that *Enterococcus*, *Pantoea*, *Incertae*_*Sedis*, *Escherichia*-*Shigella*, *Anderseniella* and *Acinetobacter* were sensitive to transient high temperature (Table 4).

**Table 4:** Proportion of genera in the intestinal bacterial community at different time points in all samples.

No. Genus	CT-48	CT-96	CT-144	HT-48	HT-96	HT-144
1 *Lactococcus*(+)	25.99	19.3	17.73	30.54	22.76	30.01
2 *Enterococcus*(+)	0.34	30.71	48.99	0.31	0.23	3.97
3 *Pantoea*(−)	15.24	0.25	0.04	0.29	0.18	0.08
4 *Bacillus*(+)	7.68	5.72	5.02	9.37	7.66	8.43
5 *Pseudomonas*(−)	2.93	2.35	1.61	3.37	2.82	2.62
6 *unclassified_Ruminococcaceae*(+)	1.49	1.64	0.69	2.93	0.93	0.64
7 *Arthrobacter*(−)	2.19	1.75	1.51	2.58	2.14	2.33
8 *Solibacillus*(+)	2.03	1.4	1.31	2.65	1.99	2.22
9 *unclassified*_*Anaerolineaceae*	0.8	0.86	0.58	2.28	1.06	0.58
10 *Burkholderia* (−)	1.56	1.23	1.29	1.67	1.73	1.59
11 *nclassified*_*Porphyromonadaceae* (−)	0.92	0.67	0.38	1.57	0.5	0.32
12 *Streptococcus*(+)	1.33	1	0.8	1.61	1.18	1.36
13 *Leuconostoc*(+)	1.46	1.18	0.8	1.52	0.92	0.71
14 *Escherichia*-*Shigella* (−)	0.8	0.62	0.35	1.47	0.93	2.7
15 *Variovorax* (−)	0.78	0.46	1.14	1.35	1.27	0.86
16 *Alistipes* (+)	1.4	1.2	0.66	2.37	0.58	0.39
17 *Erythrobacter* (−)	0.93	0.51	0.49	1.27	0.8	0.59
18 *Lactobacillus* (+)	0.9	0.62	0.41	1.16	0.77	0.5
19 *Allobaculum*	0.92	0.88	0.51	1.09	0.54	0.42
20 *Incertae*_*Sedis*	0.71	0.54	0.31	0.85	0.58	8.33
21 *Staphylococcus*(+)	0.73	1.71	2.56	0.6	2.03	2.42
22 *Anderseniella*(−)	3.66	1.12	0.59	0.56	0.62	9.17
23 *Acinetobacter*(+)	2.68	1.48	0.25	0.45	0.30	2.44

+, Gram-positive bacterium; −, Gram-negative bacterium.

### Similarity of the intestinal flora in the control and THTT samples

**Figure 5:**
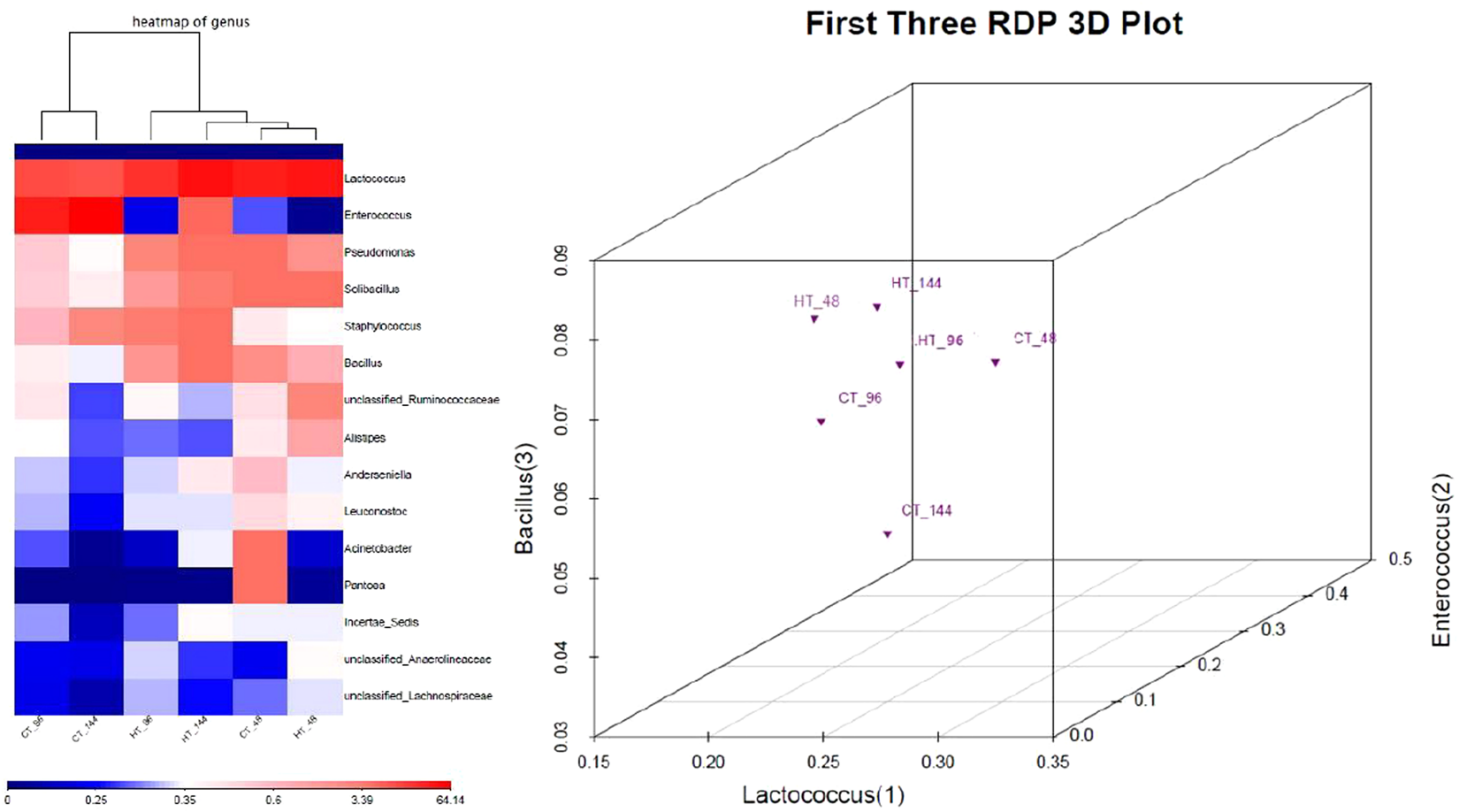
Heatmap and RDP 3D plot of the different samples. (**a**) A heatmap of bacterial communities in the CT-48, CT-96, CT-144, HT-48, HT-96 and HT-144 samples based on the top 15 dominant genera. Rows and columns represent the samples and dominant genera, respectively. The color blocks in the heat map represent weighted-UniFrac distances, and the lower number represents greater similarity in bacterial microbiota between samples in the heatmap. The deeper red color indicates a closer distance between samples, and the deeper blue color indicates a larger distance between samples. (**b**) A RDP 3D plot of bacterial communities in the CT-48, CT-96, CT-144, HT-48, HT-96 and HT-144 samples based on the top 3 dominant genera (*Enterococcus*, *Lactococcus*, *Pseudomonas*). Closer spatial distances indicate more similarity between the samples.

The top 15 dominant genera were selected, and the similarity of microbial communities in the CT-48, CT-96, CT-144, HT-48, HT-96 and HT-144 samples was assessed using a distance matrix based on weighted-UniFrac distances. A heatmap displaying the similarity of bacterial populations in different samples was generated (Fig. 5a). The samples could be divided into two categories as follows: one class consisted of HT-48, CT-48, HT-96 and HT-144 with CT-48 and HT-48 being highly similar; and the other class consisted of CT-96 and CT-144. These categories indicated that the silkworm intestinal bacterial population was sensitive to the surrounding temperature and significantly changed as the larvae matured.

According to the previous report of Xiao *et al*.^32^, we selected the top 3 dominant genera (*Lactococcus*, *Enterococcus* and *Bacillus*) among all samples to further assess the similarity of bacterial communities using PCoA. The results showed that the samples were located at different positions in the RDP 3D plot (Fig. 5b) and that 3 THTT samples (HT-48, HT-96 and HT-144) were proximal in location, indicating that these three genera were significantly affected after the silkworms were exposed to transient high temperature.

### Phylogenetic tree of predominant genera

**Figure 6:**
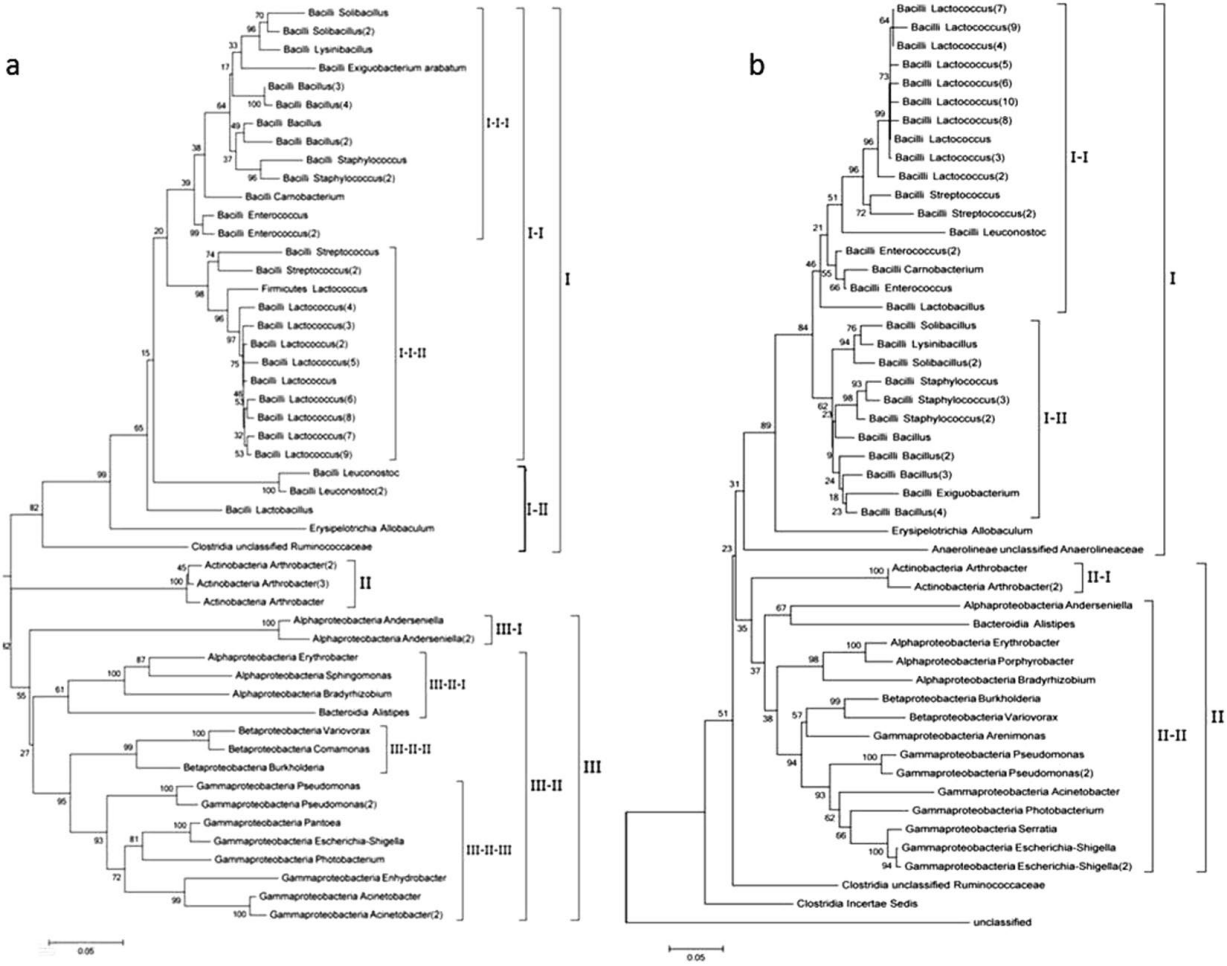
Phylogenetic tree of top 50 dominant OTUs. The phylogenetic tree was constructed using the Maximum Likelihood method. (**a**) Control samples; (**b**) THTT samples.

Based on the clustering results, representative OTU sequences with the highest abundance in each OTU cluster of the abundance top 50 OTU clusters were used to construct a phylogenetic tree through the maximum likelihood method to understand the evolutionary relationships among the silkworm intestinal bacteria. In the control samples, the OTUs were grouped into 3 categories (I, II and III) as follows: category I was composed of Bacilli; category II consisted of *Arthrobacter*, which belong to Actinobacteria; and category III consisted of Proteobacteria, including Alphaproteobacteria, Betaproteobacteria and Gammaproteobacteria. Category I was further divided into 2 subcategories (I-I and I-II), and subcategory I-I was composed of branch I-I-I with *Enterococcus*, *Bacillus*, *Staphylococcus*, *Solibacillus*, *Lysinibacillus*, *Exigucbacterium* and *Carnobacterium* and branch I-I-II with *Streptococcus* and *Lactococcus*. Category III was divided into two subcategories as follows: III-I (*Anderseniella*) and III-II (Alphaproteobacteria, Betaproteobacteria and Gammaproteobacteria) (Fig. 6a). In the THTT samples, the OTUs were divided into two categories (I and II) (Fig. 6b). Category I mainly consisted of Bacilli, which was further divided into two subcategories as follows: subcategory I-I with *Lactococcus*, *Streptococcus*, *Enterococcus*, *Leuconostoc*, *Carnobacterium* and *Lactobacillus*; and subcategory I-II with *Solibacillus*, *Lysinibacillus*, *Staphylococcus*, *Bacillus* and *Exigucbacterium*. Category II was divided into two subcategories (II-I and II-II) as follows: II-I consisted of *Actinobacteria*; and II-II consisted of *Anderseniella*, *Alistipes*, *Erythrobacter*, *Porphyrobacter*, *Bradyrhizobium*, *Variovorax*, *Aeromonas*, *Pseudomonas*, *Acinetobacter*, *Photobacterium*, *Serratia* and *Escherichia*-*shigella*. It is worth mentioning that the majority of OTUs were assigned to *Bacillus*, *Pseudomonas* and *Serratia*, which were all among the top 50 pathogens in the THTT samples, whereas only *Bacillus* was among the top 50 pathogens in the control sample.

### Interaction of silkworm intestinal bacteria

**Figure 7:**
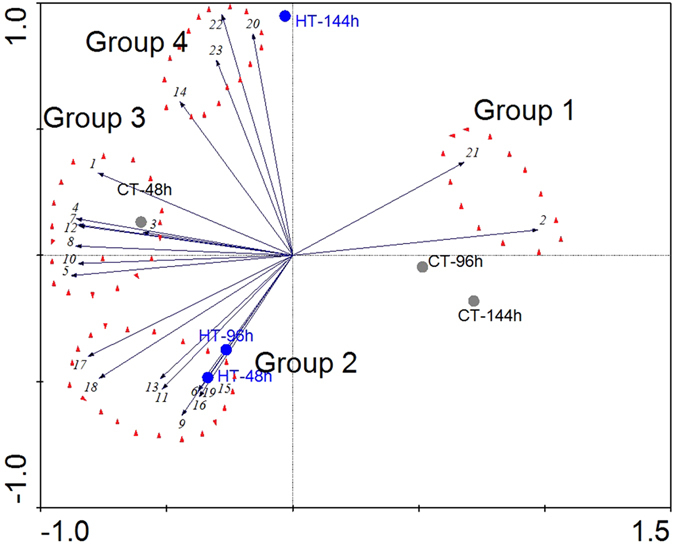
PCA analysis of the predominant genera according to their abundance in the intestinal microbiota. 1–23 represent dominant genera mentioned in Table 4.

To further understand the interaction and effects of transient high temperature on silkworm intestinal bacteria, the relevance of abundance of the predominant genera (Table 4) in gut flora was assessed using species matrix analysis. The dominant bacteria were located in three quadrants (Fig. 7) and divided into four groups (Group 1, Group 2, Group 3, and Group 4). A positive correlation was found among the abundance of bacteria in the same Group. The abundance of *Enterococcus* (2) and *Staphylococcus* (21) in Group 1 was negatively correlated with the abundance of bacteria in Groups 2, 3 and 4. The abundance of bacteria in Group 3 showed a positive correlation with the abundance of bacteria in Groups 2 and 4, but the abundance of bacteria in Group 2 showed no significant relevance with that in Group 4. Individual samples were distributed to different locations (Fig. 7). CT-48 was distinctly clustered away from CT-96 and CT-144, implying a change in the bacterial composition over time and suggesting that the change in the treated samples may not be due to increased temperature alone. The HT-48 and HT-96 samples were gathered together, and the abundance of bacteria in Group 2 became substantially greater in the two samples after the silkworms were exposed to transient high temperature. The abundance of bacteria in Group 4 increased after the THTT-silkworms were reared at normal temperature for 144 h (HT-144). The abundance of bacteria in Group 2 decreased after the silkworms were subjected to THTT (Fig. 7).

### Effect of THTT on gene expression in the silkworm midgut

**Figure 8:**
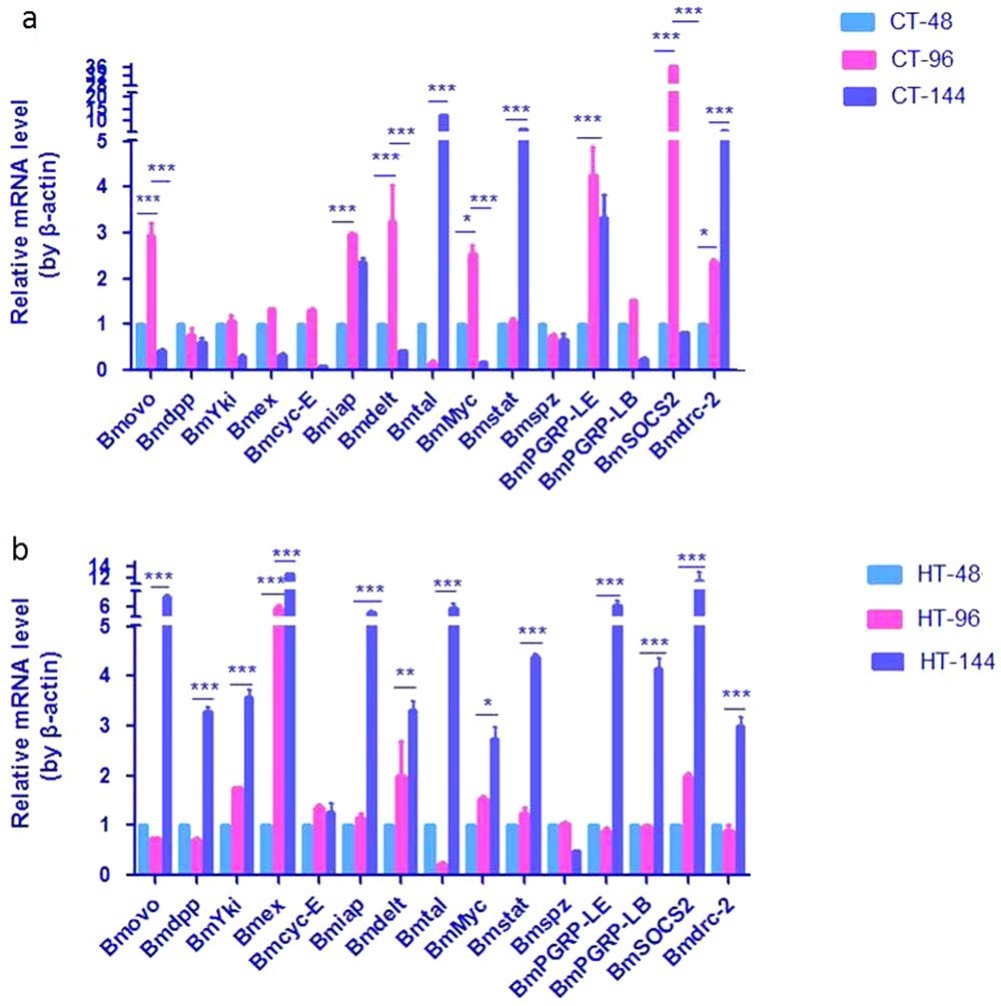
Relative expression levels of genes in the midgut of the fifth instar larvae in all samples. (**a**) Control samples; (**b**) THTT samples; CT/HT-N, samples mentioned in Table 1.

To understand the effects and mechanism of high temperature on silkworm intestinal bacteria, the relative expression levels of genes related to innate immunity, cell cycle and apoptosis were determined by qRT-PCR. During maturity of the fifth instar silkworm larvae, there were no significant changes in the midgut of control samples for the following genes: decapentaplegic (*Bmdpp*: GenBank accession No. KP861420), yorkie (including *Bmyki*-1: GenBank accession No. KF904339 and *Bmyki*-3: GenBank accession No., KF904311), expanded (*Bmex*: GenBank accession No. XM_012693250), Cyclin E (*Bmcyc-E*: GenBank accession No. AB457002), spatzle-1 (*Bmspz*: GenBank accession No. NM_001114594) and peptidoglycan-recognition protein LB (*BmPGRP-LB*: GenBank accession No. XM_012692645). In the midgut, the gene expression level changes were similar for the following genes: Ovo 1 (*Bmovo*: GenBank accession No. GU477588), Delt (*Bmdelt*: GenBank accession No. NM_001163899), c-myc (*Bmmyc*: GenBank accession No. NM_001257008) and suppressor of cytokine signaling (*BmSOCS*2: GenBank accession No. HQ540306). The expression levels of these genes increased in CT-96 and decreased in CT-144. The expression level of dicer-2 (*Bmdrc*-2: GenBank accession No. NM_001193614) was increased with maturity of the fifth instar larvae. Additionally, the gene expression levels of the inhibitor of apoptosis protein (*Bmiap*: GenBank accession No. NM_001043559) and peptidoglycan-recognition protein LE (*BmPGRP-LE*: GenBank accession No. XM_004929909) were highest in CT-96, and there was no significant difference observed between CT-48 and CT-144. The expression levels of tarsal-less (*Bmtal*: GenBank accession No. NM_001099847) and signal transducer and activator of transcription (*Bmstat*: GenBank accession No. NM_001163916) were highest in CT-144, and no significant differences were recorded between CT-48 and CT-96. These results indicated that the expression level changes of the genes related to innate immunity, cell cycle and apoptosis were different during the development of the fifth instar silkworm larvae (Fig. 8a).

To understand the effect of transient high temperature on gene expression, the expression levels of the abovementioned genes in the HT-48, HT-96 and HT-144 samples were determined by qRT-PCR (Fig. 8b). The results showed that there were no significant differences in the expression levels of *Bmcyc*-E and *Bmspz* in the HT-48, HT-96 and HT-144 samples. The gene expression levels of *Bmovo*, *Bmdpp*, *Bmyki*, *Bmiap*, *Bmdelt*, *Bmtal*, *BmMyc*, *BmSTAT*, *BmPGRP-LE*, *BmPGRP-LB*, *BmSOCS*2 and *Bmdrc*-2 were not significantly different between the HT-48 and HT-96 samples, but the highest expression levels were recorded in the HT-144 sample. The gene expression levels of *Bmex* in the HT-96 and HT-144 samples were increased by 7 and 14 times, respectively, compared to the HT-48 sample.

**Figure 9:**
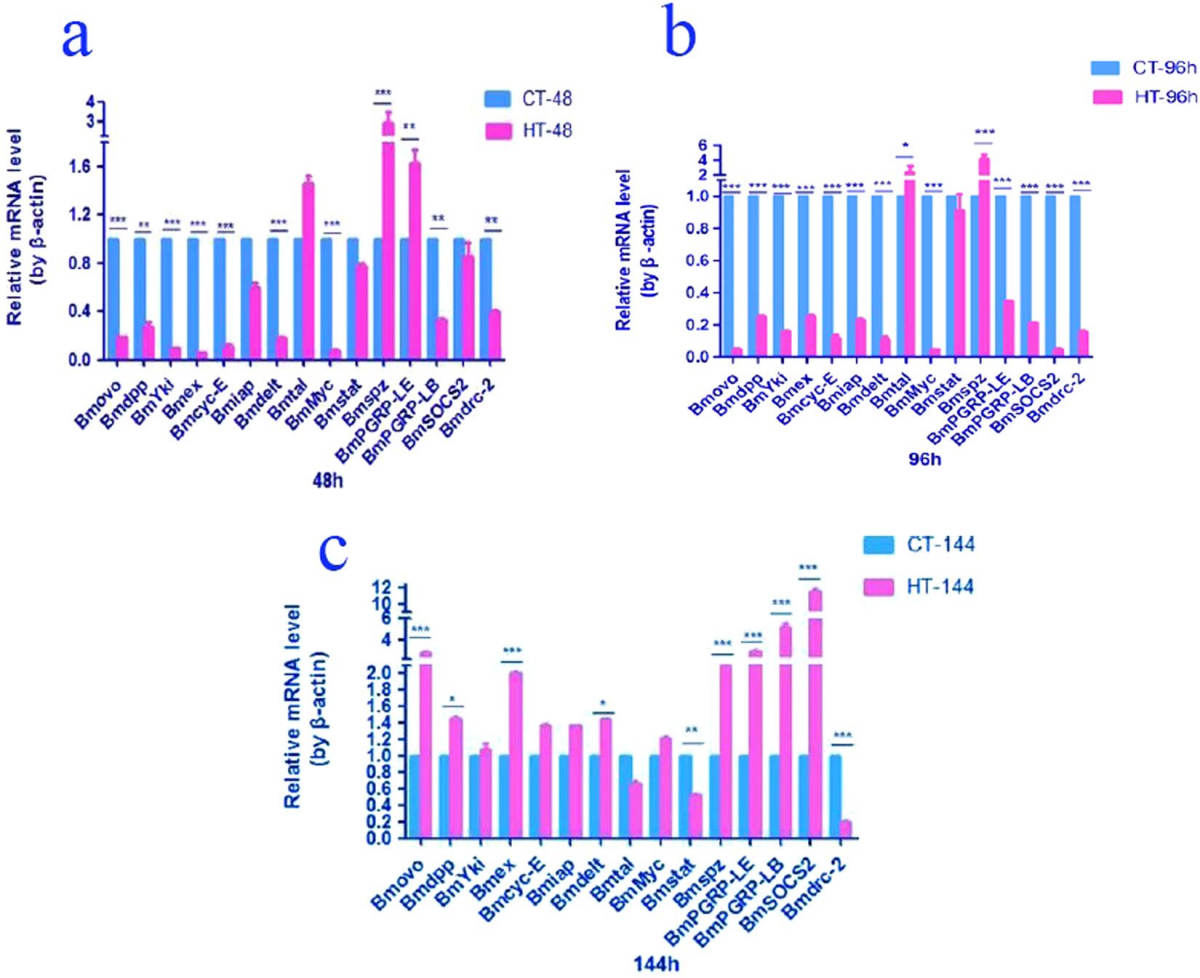
Comparison of gene expression levels in the midgut between control and THTT silkworms.

With regard to *Bmspz* and *BmPGRP*-*LE*, the expression levels were increased by 2.9 and 1.6 times, respectively, in the HT-48 sample compared to the corresponding CT-48 control, but the expression levels of *Bmovo*, *Bmdpp*, *Bmyki*, *Bmex*, *Bmcyc*-E, *Bmiap*, *Bmdelt*, *Bmmyc*, *BmPGRP*-*LB* and *Bmdrc*-2 were decreased. The expression levels of *Bmtal*, *Bmstat* and *BmSOCS*2 did not change in the HT-48 and CT-48 samples (Fig. 9a).

In the HT-96 sample, the expression levels of *Bmtal* and *Bmspz* were higher than those in CT-96, and *Bmstat* showed similar gene expression levels in both the HT-96 and CT-96 samples. The expression levels of other detected genes in the HT-96 sample were lower than those in the CT-96 sample (Fig. 9b).

In the HT-144 sample, the expression levels of *Bmovo*, *Bmex*, *Bmspz*, *BmPGRP*-*LE*, *BmPGRP*-LB and *BmSOCS*2 were increased significantly by 2.9, 2, 2.1, 3, 5.2 and 11.6 times, respectively, compared to the corresponding control (CT-144). *Bmdpp* and *Bmdelt* also showed increased expression levels in the HT-144 sample compared to the control, while the expression levels of *Bmstat* and *Bmdrc*-2 in the HT-144 sample were lower than those in the CT-144 sample (Fig. 9c).

### Relationship of gene expression levels and dominant bacterial genera

To explore the relationships among the THTT, intestinal flora and the expression of genes related to innate immunity, cell cycle and apoptosis in silkworm, the abundance of dominant bacterial genera (shown in Table 4) and gene expression level data (−∆Ct value) were used for gene expression-species matrix analysis. First, detrended correspondence analysis (DCA) was performed, and redundancy analysis (RDA) was used to address the connection between microbial communities and gene expression level because the gradient lengths were less than 3.

**Figure 10:**
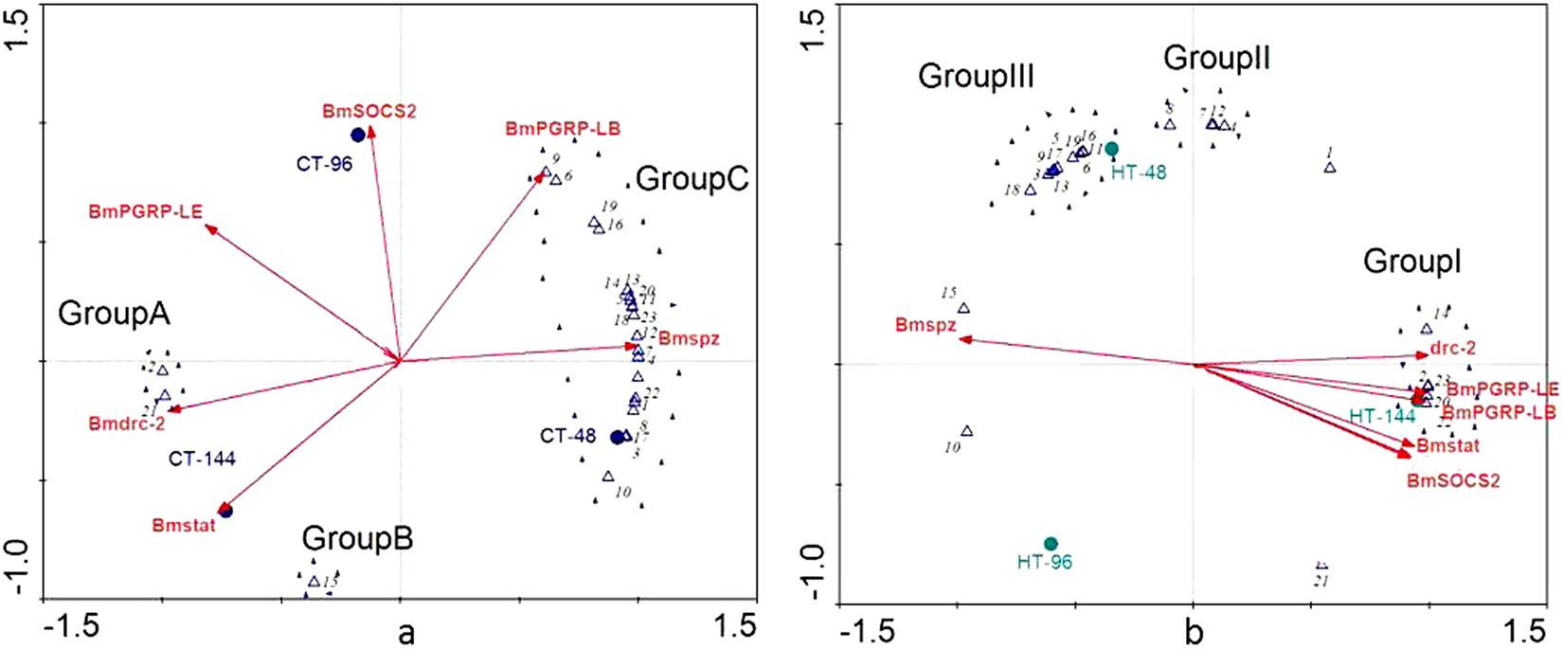
Correlation analysis of intestinal bacteria and gene expression. (**a**) Control silkworms; (**b**) THTT silkworms. The numbers represent bacterial genera mentioned in Table 4.

The results showed that the dominant bacterial genera in the gut content of the control silkworm were divided into three groups. *Enterococcus* (2) and *Staphylococcus* (21) were assigned into Group A, and their abundances were positively correlated. *Variovorax* (15) was assigned to Group B, and other dominant bacteria (Table 4) were assigned to Group C (Fig. 10a). The expression level of *Bmspz* was negatively correlated with the abundance of bacteria in Group A and positively correlated with the abundance of bacteria in Group C, but *Bmdrc*-2 showed an opposite trend. The abundance of bacteria in Group B was positively correlated with the gene expression level of *Bmstat* but negatively correlated with the gene expression levels of *BmSOCS*2 and *BmPGRP*-LE.

The distribution of the dominant bacterial genera in the midgut content of the THTT silkworm was dispersed (Fig. 10b). *Enterococcus* (2), *Escherichia*-*Shigella* (14), *Incertae*_*Sedis* (20), *Anderseniella* (22) and *Acinetobacter* (23) were distributed in the HT-144 sample (Group I). *Bacillus* (4), *Arthrobacter* (7), *Solibacillus* (8), and *Streptococcus* (12) were assembled to form Group II. *Pantoea* (3), *Pseudomonas* (5), *unclassified*_*Ruminococcaceae* (6), *unclassified*_*Anaerolineaceae* (9), *unclassified*_*Porphyromonadaceae* (11), *Leuconostoc* (13), *Alistipes* (16), *Erythrobacter* (17), *Lactobacillus* (18) and *Allobaculum* (19) were assigned to Group III, and their abundance was relatively higher in the HT-48 sample compared to the other samples. *Lactococcus* (1), *Burkholderia* (10), *Variovorax* (15) and *Staphylococcus* (21) were dispersed in the ordination plot (Fig. 10b). The gene expression level of *Bmspz* was positively correlated with *Variovorax* (15) in the THTT silkworm but negatively correlated with bacteria in Group I. The abundance of bacteria in Group I was positively correlated with the expression levels of *Bmdrc*-2, *BmPGRP*-*LE*, *BmPGRP*-*LB*, *Bmstat* and *BmSOCS*2, and the abundance of bacteria in Group III was negatively correlated with the expression levels of the abovementioned genes. After exposure of silkworms to transient high temperature, the association pattern of gene expression was slightly changed, and *Bmspz* gene expression was negatively correlated with other detected genes related to innate immunity.

## Discussion

Previous studies have indicated that the structure of insect intestinal flora is closely related to the environment, food, development stage and physiological status^33^. Silkworms exposed to high temperature and humidity show decreased pathogen resistance, which often leads to the occurrence of intestinal bacterial and viral diseases, resulting in decreased cocoon yield and quality^1^. To understand the effect of high temperature stress on the intestinal flora and the relationship between gene expression and intestinal flora, we investigated the alterations of gut flora through high-throughput sequencing based on the 16 S rRNA gene after transient high temperature treatment of silkworms. Subsequently, the expression levels of genes related to innate immunity, cell cycle and apoptosis were also determined in the midgut by qRT-PCR.

According to 16 S rRNA gene sequencing results, the numbers of bacteria in the gut content of the silkworm at the genus and species levels gradually decreased with the development or maturity of fifth instar larvae. We reported a similar trend in our previous report^20^. The silkworms were treated with transient high temperature followed by rearing at normal temperature (25 °C) for 48 h (HT-48), 96 h (HT-96) and 144 h (HT-144). The numbers of bacteria at the genus (species) level in HT-96 and HT-144 samples were 165 (204) and 137 (176), respectively, which were higher than the corresponding controls. However, the HT-48 sample had less bacteria (11) at the species level than the corresponding control and more bacteria (38) at the genus level than the corresponding control. These results indicated that the diversity of intestinal bacteria increased after the THTT silkworms were reared at normal temperature. The increase range depended on the length of time (48, 96, and 144 h) that the THTT silkworms were reared at normal temperature (25 °C) after treatment. The bacterial diversity was increased after the THTT silkworms were reared at normal temperature (25 °C) for 96 h, and it decreased after 144 h, suggesting that the influence of THTT on the intestinal bacterial diversity was delayed and that the effect of high temperature was reduced with passage of time or as the silkworms matured. The THTT not only affected intestinal bacterial diversity but also influenced the abundance of intestinal bacteria. The richness of the predominant bacterial genus *Enterococcus* in the HT-48 sample was similar to that in the corresponding control (CT-48), but the abundance of *Enterococcus* in the HT-96 and HT-144 samples was decreased by 133.4 and 12.18 times, respectively, compared to corresponding controls. For the CT-96 and CT-144 samples, the community richness of *Enterococcus* was increased by 89.3 and 143.1 times, respectively, compared to the CT-48 sample, and the same richness in the HT-96 and HT-144 samples was 0.74 and 12.8 times higher, respectively, than the HT-48 sample. Therefore, the abundance of *Enterococcus* was decreased by THTT, but the effect decreased after the THTT silkworms were reared at normal temperature for 96 to 144 h. In contrast, the abundance of *Lactococcus* and *Bacillus* increased after the silkworms were exposed to high temperature, suggesting that the abundance response of gut bacteria to THTT depended on the type of bacteria. The abundance changes of *Enterococcus*, *Incertae_Sedis*, *Escherichia-Shigella*, *Anderseniella* and *Acinetobacte*r were significant, suggesting that these bacterial genera are sensitive to high temperature.

Bacterial diseases often caused by intestinal flora imbalance and secondary bacterial septicemia of the silkworm can be induced by high temperature, which may be linked to changes in the gut flora, thus decreasing resistance or immunity^34–36^. *Bacillus*, *Pseudomonas* and *Serratia* are opportunistic pathogens^37, 38^ to the silkworm, and the occurrence of secondary bacterial septicemia in silkworm is often induced by high temperature and has a high impact on cocoon production and economic loss.

To investigate the effect and mechanism of high temperature treatment on intestinal bacterial population, the expression level changes of genes related to the immune pathway, apoptosis and cell cycle were determined by qRT-PCR after THTT. The results showed that when THTT silkworms were reared at normal temperature for 48 h, the gene expression levels in the midgut were decreased compared to the corresponding controls, except for *Bmtal*, *Bmspz* and *BmPGRP-LE* genes, indicating that the gene expression levels varied in response to high temperature^39, 40^. *Bmspz* and *BmPGRP-LE*/*LB* are related to the Toll and IMD signaling pathways, respectively^28^. The gene expression levels of *Bmspz* and *BmPGRP-LE* were increased in HT-48, indicating that the Toll and IMD pathways were activated after the silkworms were exposed to high temperature. The expression level of *BmPGRP*-LE in HT-96 was lower than that of the corresponding control, and the gene expression level of *Bmspz* was higher than that of the corresponding control, suggesting that the IMD signaling pathway was inhibited and that the Toll signaling pathway was activated. These results indicated that the responses of innate immunity pathways to high temperature are different. The gene expression levels of *Bmspz*, *BmPGRP-LE* and *BmPGRP*-*LB* in HT-144 were elevated compared to CT-144. The abundance and diversity of intestinal bacteria in HT-144 were higher than those in CT-144. The antimicrobial peptide gene expression is controlled by the Toll and IMD pathways^44, 42^. In general, activation of the Toll and IMD pathways is associated with the expression of *Bmspz* and *BmPGRP* genes, which leads to the expression of antimicrobial peptide^28^. Therefore, we suggested that high temperature can modulate the Toll and IMD pathways, which lead to change in intestinal bacterial population via regulating antimicrobial peptide gene expression.

In the HT-48 sample, the *Bmstat*, *Bmdrc*-2 and *Bmdpp* genes were associated with the JAK/STAT pathway^43, 44^, RNAi pathway^28^ and TGF-β pathway^45^. Moreover, two genes, namely, *Bmyki* and *Bmex*, and three downstream genes, *Bmcyc-E*, *Bmiap* and Bmmyc, were related to the Hippo pathway^46^, and the *Bmovo* gene was associated with ovarian development^47, 48^. The expression levels of the abovementioned genes were decreased in the HT-48 sample compared to the CT-48 sample. The JAK/STAT pathway is an important natural immune pathway in the silkworm, and it responds specifically to virus infection^43^. The JAK/STAT pathway genes in the silkworm gut may be activated by oral infection of *Escherichia coli* bacteria^49^. In the present study, we found that the abundance of *Escherichia shigella* in HT-48, HT-96 and HT-144 samples was higher than that in the corresponding controls. Among the THTT silkworms, the abundance of *Escherichia shigella* was higher in HT-144 followed by HT-48 and HT-96 samples. However, the JAK/STAT pathway was inhibited in the HT-144 sample, suggesting that inhibition of the JAK/STAT pathway was attributed to high temperature as the increase in temperature resulted in a change in bacterial composition and inhibition of the JAK/STAT pathway.

The *Bmdpp* gene is related to the TGF-β pathway and is involved in immune response^44^. Our previous study revealed that the expression level of *Bmdpp* gene is enhanced by injecting inactivated *Bacillus bombysepticus* into silkworms^44^. The abundance of *Bacillus* in the THTT silkworms was increased compared to the control silkworms, indicating that the increase in the proportion of *Bacillus* was due to increased temperature. However, *Bmdpp* gene expression decreased. Thus, it could be considered that the decline in the *Bmdpp* gene expression level was not caused by *Bacillus* but was instead triggered by high temperature.

According to a previous report, cell-specific loss of the miRNA-processing enzyme, Dicer, leads to a reduction of miRNA abundance in mouse feces, which results in uncontrolled gut microbiota and exacerbated colitis. Bacterial gene transcripts can be specifically regulated by miRNAs from intestinal epithelial cells and Hopx-positive cells, which affect bacterial growth^50^. In our experiment, the gene expression of *Bmdrc*-2 in the midgut was decreased after treatment of silkworms with transient high temperature. It remains unknown whether the intestinal microbial populations in the silkworms are related to the *Bmdrc*-2 gene; thus, this relationship requires further study.

Our earlier reports indicated that the development of oocytes and ovaries may be regulated by the *Bmovo* gene^47, 48^. In the current study, the expression levels of *Bmovo* in the HT-48 and HT-96 samples were higher than those in corresponding controls, and the expression level of *Bmtal*, which regulates the gene expression of *Bmovo*, was higher than that in control samples, suggesting that the development of oocytes and ovaries may be altered by high temperature. The expression of level of *Bmovo* in the HT-144 sample was greater than that in the CT-144 sample, which may be associated with ovary development compensation. The germ line of *D*. *melanogaster* has been reported to be influenced by gut bacteria, and the main impact on oogenesis is related to the lack of *Acetobacter* ^16^. It is unknown whether gut bacteria can affect oogenesis in the silkworm through alteration of expression levels of genes related to oocyte development, indicating that further investigations should be performed.

The control of the cell cycle and cell size is associated with the Hippo pathway^46^. To meet the needs of increased appetite, midgut epithelial cells and midgut are quickly enlarged in the fifth instar larvae. In this study, we found that the expression levels of *Bmyki*, *Bmex*, *Bmmyc*, *Bmiap* and *Bmcyc*-E genes, which are related to the Hippo pathway, in HT-48 and HT-96 were lower than the levels in the respective controls, indicating that the Hippo pathway is repressed by high temperature, thereby leading to the suppression of midgut development and ultimately affecting the intestinal bacterial population through change in intestinal physiological status.

The Notch pathway is involved in cell differentiation, apoptosis and proliferation^51^, and *Bmdelt* is a ligand of the Notch pathway. Inhibitors of apoptosis comprise a highly conserved family of endogenous antiapoptotic factors. In this study, we found that gene expression levels of *Bmdelt* and *Bmiap* in HT-48 and HT-96 were lower than those in the control, but the levels in HT-144 showed an opposite trend. Therefore, the expression levels of genes related to cell proliferation and apoptosis were altered with temperature change, thus leading to change in intestinal homeostasis and ultimately affecting the intestinal bacterial population.

Interaction among the silkworm intestinal bacteria has been indicated in our previous work^20^. In our current study, the abundance of *Enterococcus* (2) and *Staphylococcus* (21) in Group 1 showed a negative correlation with the abundance of bacteria in Groups 2, 3 and 4 (Fig. 7). The bacteria in Groups 3 and 2 were positively associated with the bacteria of Group 4, and the abundance of bacteria in Group 2 showed no significant relevance with Group 4. These interactions were slightly different from the results found in intestinal bacteria of BmCPV-infected silkworms^20^, which indicated that high temperature treatment also affects the interaction of intestinal bacteria.

To explore the correlation of dominant bacterial genera in intestinal bacterial population with host gene expression, the correlation between the abundance of dominant genera and the expression level of genes in all samples were analyzed. In control samples, the abundance of *Enterococcus* (2) and *Staphylococcus* (21) (Group A) demonstrated a positive correlation with the expression level of *Bmdrc*-2 (RNAi pathway) and a negative correlation with the expression level of *Bmspz* (Toll pathway). However, other dominant bacteria (Group C) showed opposite trends with the expression levels of these genes. In the THTT samples, the abundance of *Variovorax* (15) positively correlated with the expression level of *Bmspz* and negatively correlated with the expression levels of *Bmdrc*-2, *BmPGRP*-*LE*, *BmPGRP*-*LB*, *Bmstat* and *BmSOCS*2. The abundance of *Enterococcus* (2), *Escherichia-Shigella* (14), *Incertae_Sedis* (20), *Anderseniella* (22) and *Acinetobacter* (23) in Group II was positively correlated with the expression levels of *Bmdrc*-2 (RNAi pathway), *BmPGRP-LE* and *BmPGRP-LB* (IMD pathway). These outcomes revealed that high temperature impact not only changes the expression pattern of genes but also changes the impact pattern of gene expression on intestinal bacteria. The correlation changes of the related genes between the control and THTT silkworms demonstrated that high temperature also affects interaction of genes. Therefore, we concluded that there is a direct connection among the THTT, intestinal flora and host gene expression in intestinal gut flora of the silkworm.

## Electronic supplementary material

Table S1
