# Supplementary material for: Effects of transient high temperature treatment on the intestinal flora of the silkworm Bombyx mori
Source: Sci Rep. 2017 Jun 13;7:3349. doi: 10.1038/s41598-017-03565-4 (PMC5469737; doi:10.1038/s41598-017-03565-4)
Supplement: Supplementary file 1 — Table S1 [file 41598_2017_3565_MOESM1_ESM.docx]

| Samples | GenBank accession No. |
| --- | --- |
| CT-48-1 | SRR3347781 |
| CT-48-2 | SRR3347782 |
| CT-96-1 | SRR3347829 |
| CT-96-2 | SRR3347830 |
| CT-144-1 | SRR3347832 |
| CT-144-2 | SRR3347833 |
| HT-48-1 | SRR3347763 |
| HT-48-2 | SRR3347765 |
| HT-96-1 | SRR3347769 |
| HT-96-2 | SRR3347774 |
| HT-144-1 | SRR3347775 |
| HT-144-2 | SRR3347780 |

**Effects of transient** **high temperature treatment on** **the intestinal flora of the silkworm *Bombyx mori***

Zhenli Sun^1^*, Dhiraj Kumar^1^*, Guangli Cao^1,2,3^, Liyuan Zhu^1^, Bo Liu^1^, Min Zhu^1^, Zi Liang^1^, Sulan Kuang^1^, Fei Chen^1^, Yongjie Feng^1,2,3^, Xiaolong Hu^1,2,3^, Renyu Xue^1,2,3^, Chengliang Gong^1,2,3#^

1 School of Biology & Basic Medical Science, Soochow University, Suzhou, 215123, China,

2 National Engineering Laboratory for Modern Silk, Soochow University, Suzhou, 215123, China

3 Agricultural Biotechnology Research Institute, Agricultural biotechnology and Ecological Research Institute, Soochow University, Suzhou, 215123, China,

*These authors contributed equally to this work.

# Corresponding author

Tel.: +86-521-65880183; Fax: +86-521-65880183.

E-mail addresses: gongcl@suda.edu.cn

Postal address: Pre-clinical Medical and Biological Science College,

Soochow University,

No.199 Ren-ai Road

Suzhou, Jiangsu, China

Table S1 The GenBank accession number of the original data of Illumina miseq sequencing
